# The Modulatory Influence of Plant-Derived Compounds on Human Keratinocyte Function

**DOI:** 10.3390/ijms222212488

**Published:** 2021-11-19

**Authors:** Anna Merecz-Sadowska, Przemysław Sitarek, Karolina Zajdel, Ewa Kucharska, Tomasz Kowalczyk, Radosław Zajdel

**Affiliations:** 1 Department of Computer Science in Economics, University of Lodz, 90-214 Lodz, Poland; radoslaw.zajdel@uni.lodz.pl; 2 Department of Biology and Pharmaceutical Botany, Medical University of Lodz, 90-151 Lodz, Poland; przemyslaw.sitarek@umed.lodz.pl; 3 Department of Medical Informatics and Statistics, Medical University of Lodz, 90-645 Lodz, Poland; karolina.smigiel@umed.lodz.pl; 4 Chair of Gerontology, Geriatrics and Social Work at the Faculty of Pedagogy, Ignatianum Academy in Cracow, 31-501 Cracow, Poland; ewa.kucharska@vadimed.com.pl; 5 Department of Molecular Biotechnology and Genetics, University of Lodz, 90-237 Lodz, Poland; tomasz.kowalczyk@biol.uni.lodz.pl

**Keywords:** keratinocytes, plants, secondary metabolites, ROS, inflammation, UV radiation, wound healing

## Abstract

The plant kingdom is a rich source of secondary metabolites with numerous properties, including the potential to modify keratinocyte biology. Keratinocytes are important epithelial cells that play a protective role against various chemical, physical and biological stimuli, and participate in reactive oxygen scavenging and inflammation and wound healing processes. The epidermal cell response may be modulated by phytochemicals via changes in signal transduction pathways. Plant extracts and single secondary compounds can possess a high antioxidant capacity and may suppress reactive oxygen species release, inhibit pro-apoptotic proteins and apoptosis and activate antioxidant enzymes in keratinocytes. Moreover, selected plant extracts and single compounds also exhibit anti-inflammatory properties and exposure may result in limited production of adhesion molecules, pro-inflammatory cytokines and chemokines in keratinocytes. In addition, plant extracts and single compounds may promote keratinocyte motility and proliferation via the regulation of growth factor production and enhance wound healing. While such plant compounds may modulate keratinocyte functions, further in vitro and in vivo studies are needed on their mechanisms of action, and more specific toxicity and clinical studies are needed to ensure their effectiveness and safety for use on human skin.

## 1. Introduction

Plants produce a range of secondary metabolites. These not only play a crucial role in the adaptation of plants to the environment but also have a strong impact on other living organisms, including humans. Various groups of molecules with a plant origin such as phenolics, alkaloids, saponins, terpenes, lipids and carbohydrates, are involved in a plethora of biological activities. Selected phytochemicals may modulate several cell-signaling pathways in different cell types, including those of epidermal cells [1].

The skin creates a barrier protecting the host from the outside environment. The outer layer of the human skin, the epidermis, is formed by keratinocytes. These cells are on the first line of defense against various harmful chemical, physical and biological factors and are particularly vulnerable to ultraviolet (UV)-radiation, a strong source of reactive oxygen species (ROS). In response, keratinocytes produce a range of biologically active molecules that enhance ROS scavenging, inflammatory responses and wound healing [2].

Numerous plant extracts, as well as their component compounds, exert antioxidant activity. Phenolic compounds are particularly effective ROS scavengers. These chemicals are able to neutralize ROS via their ability to donate hydrogen atoms or electrons to radicals and chelate metal cations. Selected plant extracts and their component compounds have been found to suppress ROS release, decrease the activity of inducible nitric oxide synthase and nitric oxide, inhibit pro-apoptotic proteins and apoptosis and activate antioxidant enzymes in keratinocytes following exposure to hydrogen peroxide (H_2_O_2_) [3]. A wide range of secondary metabolites is also known to play an anti-inflammatory role. Following stimulation with pro-inflammatory factors, keratinocytes produce numerous molecules including adhesion molecules, interleukins and chemokines, with this production being suppressed by exposure to plant extracts and compounds [4]. Moreover, keratinocytes treated with UV radiation are exposed to the action of both ROS and pro-inflammatory factors; indeed, selected plant extracts and single compounds may play a dual, antioxidant and anti-inflammatory role in epidermal cells exposed to UV radiation [5,6]. In addition, plant extracts and single compounds may also modulate the release of various growth factors, chemokines or neuropeptides by keratinocytes, with exposure to molecules of plant origin possibly enhancing keratinocyte migratory and proliferation rates. This effect also accelerates the wound healing process [7].

The aim of the present paper is to review the role of selected plant extracts and single compounds as modulators of keratinocyte biology, paying particular attention to stimuli, specific receptors, protein release and signal transduction pathways. It examines the potential of phytochemicals as ROS scavengers, important anti-inflammatory agents and as factors for mediating wound healing. The role, contributions and usefulness of plants were summarized.

## 2. Criteria for Paper Selection

To give an overview of the current state of knowledge regarding the topic, i.e., in vitro treatment of human keratinocytes with oxidants, pro-inflammatory agents and UV radiation followed by exposure to different plant extracts, papers were selected from those included in the electronic databases PubMed/MEDLINE, Google Scholar, Scopus and Web of Science over the past 10 years. The following search terms were used: keratinocytes, plant extract, plant-derived compounds, reactive oxygen species, inflammation, UV-irradiation and wound healing. Moreover, the impact of plant secondary compounds on keratinocyte motility and proliferation rate was evaluated. Papers reporting articles published in languages other than English, those with only an abstract or lacking full-text access, those published earlier than 10 years ago, those without identified compounds in extracts and those examining cells other than human keratinocytes were excluded. Each selected document was analyzed and the following data were extracted and presented in tables: species name, plant part, type of extract, type of cell lines, identified compounds, mechanism of action and final effect. The main text includes a characterization of the signaling cascades involved in keratinocyte biology.

## 3. Plant Secondary Metabolites

The plant kingdom is a rich source of secondary metabolites. Over 50,000 molecules have been discovered [8]. These metabolites can be divided into the following structural classes: phenolics, alkaloids, saponins, terpenes, lipids and carbohydrates [9]. These compounds play an essential role for the plant itself by allowing adaptation to the environment. Phytochemicals contribute to perennial growth, deciduous behavior, flowering, fruit set and abscission, and are known to demonstrate antimicrobial properties [8]. Many of these compounds also exhibit antioxidant [10], anti-inflammatory [11], anticancer [12], including against keratinocyte carcinomas [13], antipathogenic [14,15] or antiobesity [16] properties; as such, they are widely used in various industrial sectors, including pharmaceutical, cosmetic or food [17]. In addition, there is a strong trend towards using herbal formulations in health and wellness [18].

Secondary metabolites may be obtained from the leaves, roots, stems, bark and aerial parts of the plants; however, the plants require careful preparation, extraction and quantitative and qualitative determination of active compounds. The first step involves the selection of an appropriate solvent. The most popular are polar ones such as water and alcohols, intermediate polar ones such as acetone and dichloromethane, and nonpolar ones such as chloroform, n-hexane and ether. Following this, an appropriate extraction method must be identified. Various options exist including maceration, decoction, digestion, percolation, infusion, Soxhlet extraction, superficial extraction, microwave-assisted extractions or ultrasound-assisted extraction. The isolated compounds must then be purified via chromatographic methods and identified via spectroscopy [19]. However, the greatest challenges are those posed by the determination of the molecular mechanism of action and the later conduct of clinical trials where necessary [20].

## 4. Keratinocyte Characteristics

The skin is the largest organ in the human body. In addition to serving as a barrier for the inner environment, it also regulates body temperature, enhances metabolic functions and enables contact with the outer environment by its host of nerve endings.

Human skin consists of three layers: an outer stratified epithelium, a middle dermis and an underlying subcutaneous tissue. The epidermis is formed mostly (at least 80%) by keratinocytes. Newly-synthesized keratinocytes build up a supply of keratin in the cytoplasm and then undergo structural and biochemical changes until terminal differentiation and cell death. Keratinocytes play numerous roles including the creation of a physical barrier. That barrier provides protection against water loss, biological agents such as pathogens, physical agents such as UV radiation and various chemical agents. Moreover, epithelial cells accumulate melanin derived from melanocytes, migrate and proliferate to heal wounds when skin integrity is disrupted and participate in skin immunity. In addition, keratinocytes are involved in molecular interactions between various surrounding skin cells via different signal transduction pathways [21,22].

During normal epidermal differentiation, the following types of keratinocytes are distinguished: basal, spinous, granular and cornified. These types vary in their phenotypic and biochemical properties based on changes in gene expression. Many of these genes are regulated by p63 transcription factors, including keratins, involucrin and loricrin [23]. Basal cells that are in contact with the basement membrane through hemidesmosomes and focal adhesions possess mitotic activity and express keratins 5 (K5) and K14. During the progress of differentiation, keratinocytes demonstrate basal membrane detachment and loss of mitotic activity and migrate into suprabasal layers. These suprabasal cells attach to their neighboring cells through desmosomes, an attachment that must be broken during the progress of epithelization.

Spinous keratinocytes begin to express keratins K1 and K10, and this expression continues fully in the granular forms. They also express involucrin and loricrin, the precursors of cornified envelope proteins. These play a role in corneocyte formation, the final stage of differentiation preceded by loss of nuclei and organelles. The corneocytes are connected by corneodesmosomes and covered with lipid layers secured by a protein structure. These form a barrier to prevent water evaporation [24].

The differentiation process of keratinocytes is regulated by the mitogen-activated protein kinase (MAPK) pathway, which is induced by various stimuli including epidermal growth factor (EGF), tumor necrosis factor (TNF) and calcium influx [25]. The signaling transition pathway uses different isoforms of protein kinase C (PKC) [26]. In normal human epidermal keratinocytes (NHEKs), the inhibition of the MAPK pathway resulted in the decreased expression of proteins directly implicated in the differentiation process. For example, the suppression of p38 signaling resulted in the inhibition of K5, K14, ST14 transmembrane serine protease matriptase (ST14), small proline-rich protein 3 (SPRR3), serine/threonine kinase (Akt) expression and suppressed the nuclear factor of kappa in B cells (NF-κB) light polypeptide gene enhancer; suppression of c-Jun N-terminal kinase (JNK) signaling resulted in the inhibition of K14, SPRR3, Akt expression and suppression of NF-κB. In addition, the suppression of extracellular signal-regulated kinase 1/2 (ERK1/2) signaling resulted in the inhibition of SPRR3 and Akt expression [27].

Being located directly on the edge of the internal and external environment, epidermal keratinocytes are exposed to mechanical stress. Stretching the skin results in hyperproliferation via the induction of calcium influx followed by phosphorylation of essential growth-related factors, including the epidermal growth factor receptor (EGFR) [28]. Moreover, keratinocytes can also become activated in response to exposure to ROS, inflammatory factors, UV-radiation and skin barrier disruption. Such cells generally express various surface receptors which translate the stimulus into biological effects [29]. Ligand/receptor binding induces signal transduction pathways and changes in protein phosphorylation [30]. Plant-derived compounds are believed to modulate various signaling pathways in various cell lines, including keratinocytes, some of which include signaling cascades related to proliferation and apoptosis [31].

## 5. Modulatory Effect of Plant Secondary Metabolites on Keratinocytes Exposed to ROS

UV radiation is the main source of ROS in keratinocytes, with heat shock and drugs being others. Oxidative stress may be involved in the inflammatory condition and apoptosis of the skin. In addition, ROS may modulate various signaling cascades in human cells in vitro. ROS have also been found to mediate MAPK activities, and an oxidant state has been associated with an elevated level of activator protein 1 (AP-1) and NFκB in mouse keratinocytes [32]. However, there is a need to better identify the role of ROS in the signal transduction pathway using antioxidants including plant-derived compounds [33].

Many plants’ secondary metabolites, especially phenolic compounds, act as antioxidants. This has been attributed to their ability to donate hydrogen atoms or electrons to radicals to form nonreactive phenoxyl radicals or stable radical cations, respectively [34]. They are also believed to chelate metal cations. The oxidized metabolites of phenolic compounds have also been found to up-regulate the expression of antioxidant enzymes, and are believed to bestow considerable beneficial biological effects related to their antioxidant activity, including anti-inflammatory, anti-aging and anti-cancer properties [35,36]. Table 1 presents the impact of the selected plant extracts on H_2_O_2_-stimulated human keratinocytes. Cellular exposure to H_2_O_2_ is strictly connected with ROS induction; therefore, the presented phytochemicals exhibit important antioxidant properties such as suppressing ROS release, limiting damage to DNA and lipid oxidation, inhibiting iNOS and NO, decreasing the level of pro-apoptotic proteins and apoptosis and activating antioxidant enzymes.

## 6. The Modulatory Effect of Plant Secondary Metabolites on Keratinocytes Involved in the Inflammation Process Triggered by Physical, Chemical or Biological Agents

Unperturbed keratinocytes do not release inflammatory mediators; however, their expression may be enhanced by various stimuli, including UV radiation, wounding, pathogens and pathogen products. ROS play a special role in skin inflammation and may trigger a chronic skin response. Following the activation, the upregulation of the adhesion molecule, cytokine and chemokine expression is observed [47].

Adhesion molecules that belong to the immunoglobin superfamily, including intercellular adhesion molecule-1 (ICAM-1) and vascular adhesion molecule-1 (VCAM-1) play an essential role in inflammatory mechanisms. ICAM-1 is a ligand for the B-2 integrin leukocyte function-associated antigen-1 (LFA-1). This molecule promotes cellular interaction in the immune response. ICAM-1 and LFA-1 interaction is essential for T lymphocytes activation. Elevated ICAM-1 expression is observed in keratinocytes after interferon-gamma (IFN-γ) exposure. Tumor necrosis factor-α (TNF-α) and IL-17 act synergistically with INF- γ in the induction of ICAM-1 expression. VCAM-1 interacts with various integrins [48].

Cytokine action is initiated by the activation of NFκB pathways by Toll-Like Receptors (TLRs), which recognize pathogen-associated molecular patterns [49]. It has been shown that keratinocytes express various TLRs including 1–6 and 9 [50,51], indicating that the induced TLRs stimulate cytokines [52,53]. TLR ligands and cytokines mostly have overlapping or synergistic influences on keratinocytes [49]. Keratinocytes produce a number of cytokines, including interleukin (IL)-1, -6, -7, -8, -10, -12, -15, -18 and -20, and TNF-α. Keratinocytes also express various cytokine receptors including IL-1R, IL-2R, IL-4R, IL-6R, IL-10R, IL-13R, IL-17R, IL-18R, IL-20R, IL-24R and TNF-R [54]. 

IL-1, -6, -8 and TNF-α are well-known pro-inflammatory cytokines. IL-1 acts as a chemoattractant for keratinocytes [55], activates expression of keratin 6 [56], decreases adherence of selected pathogens to keratinocytes [57] and protects transformed keratinocytes from tumor necrosis factor-related apoptosis-inducing ligand (TRAIL)-induced apoptosis [58]. A study conducted on mice revealed that keratinocytes secrete high amounts of IL-1α, which is followed by a skin inflammatory response. IL-1α is essential for caspase-1 activation in an inflammasome-dependent manner. It is hypothesized that epidermal cells may play a critical role in skin immunity [59], and that IL-6 regulates normal keratinocyte growth [60]. However, a study of injury-induced keratinocyte stimulation indicated that IL-6 activity may regulate keratinocyte differentiation rather than proliferation [61]. The data indicate that IL-6 is involved in the restoration of the epidermal barrier after wounding [62]. Another cytokine, IL-8, is released by external stimuli, including UV radiation [63] and is believed to stimulate keratinocyte migration [64]. 

TNFα plays a differential role in epidermal keratinocytes. On the one hand, TNFα activates modulators of the actin cytoskeleton and integrins, regulates their degree of motility or attachment and stimulates the expression of collagen-degrading proteases and basement membrane components, followed by tissue repair. On the other hand, TNFα stimulates the nuclear translocation of NFκB. It is also believed to induce the expression of various genes, including those of cytokines, chemokines, growth factors and cell-surface receptors that may attract neutrophils, macrophages and T lymphocytes. Additionally, genes regulated by TNFα are involved in the modulation of the cell cycle and apoptosis [65]. 

IL-7 and IL-15 are involved in T cell homeostasis. IL-7 participates in the development of mature T lymphocytes in the thymus. In addition, IL-7 also induces the expression of the anti-apoptotic protein Bcl-2, followed by the survival of both naïve and memory T lymphocytes. IL-15 stimulates the proliferation of CD8+ T cells in a process independent of antigens. While both cytokines may help to defend organisms against pathogens and tumors, elevated production may be linked to several autoimmune diseases [66]. Research based on mouse keratinocytes indicates that elevated IL-7 levels can predispose to lymphoproliferative skin disease [67]. IL-7 and IL-15 production is reduced by UVB radiation [68,69].

Cytokines IL-10, IL-12 and IL-18 act as immunomodulators. IL-10 stimulates the Th2 immune response [70] and its level in keratinocytes is upregulated after exposure to UVB. Therefore, it has been hypothesized that they may play a role in immunosuppression [71]. IL-12 stimulates the Th1 immune response [72]. IL-12 is also believed to take part in limiting skin inflammation [73] and blocking the release of TNFα induced by UVB [74]. IL-18 stimulates the Th1 immune responses in collaboration with IL-12. The Th1 response is related to the host defense against pathogens by the stimulation of IFN-γ production. Without IL-12 collaboration, IL-18 induces the Th2 response [75].

IL-20 induces keratinocyte hyperproliferation and therefore may act as a modulator of skin inflammation [76]. Its release by keratinocytes may be followed by the production of various chemokines [49]. The chemokine structure is maintained by disulfide bonds formed between cysteine residues. The monomer is composed of an α-helix in the C terminus, a three-stranded β-sheet in the central region and an unstructured region in the N terminus. The chemokines can be divided into four groups based on the configuration of two cysteines closer to the N-termini: CC, CXC, CX3C and XC. Following stimulation with IFN-α, IFN-γ, IL-13, IL-17, TNF-α or IL-4, keratinocytes release a plethora of different chemokines belonging to three different classes, including CC, CXC and CX3C [77].

Plant extracts and their component compounds may modulate the inflammatory keratinocyte response in vitro via different mechanisms. Keratinocytes exposed to TNF-α or IFN-γ exhibit an elevated production of pro-inflammatory cytokines. To counteract this, treatment with plant extracts may downregulate the expression of ICAM-1, IL-1, IL-6, IL-8, TNF-α and selected chemokines, and suppress the NFκB and MAPKs pathways. Table 2 presents the impact of selected plant extracts on human keratinocytes stimulated with TNF-α or IFN-γ. As cellular exposure to pro-inflammatory agents is closely connected with induction by various cytokines or chemokines, the presented plant-derived compounds are believed to exhibit important anti-inflammatory properties.

*Ampelopsis glandulosa* [83] extract was found to inhibit TNF-α, IFN-γ, IL-4, IL-13 and IL-31 expression in the ear tissue of mice in vivo. This observation was in response to exposure to the inflammation inducers 2, 4-dinitrochlorobenzene (DNCB).

Studies based on single compounds indicate that HaCaT cells stimulated with TNF-α/IFN-γ exhibit a decreased IL-6, IL-8, ICAM-1 and selected chemokine production, suppression of NFκB translocation to the nucleus and lowered p38 and ERK1/2 activity following treatment with selected compounds derived from *Cudrania tricuspidate*: dihydrokaempferol, steppogenin, cudraflavanone D, cudraflavanone B, cudraflavone C, kuwanon C, cudraxanthone L, macluraxanthone B, 1,6,7-trihydroxy-2-(1,1-dimethyl-2-propenyl)-3- methoxyxanthone, cudratricusxanthone L, cudracuspixanthone A [93]. Elsewhere HaCaT keratinocytes stimulated with LPS demonstrated reduced expression of chemokine MCP-1 following exposure to damsin isolated from *Ambrosia arborescens* [94]. In addition, human keratinocytes stimulated with TNF-α/IFN-γ exhibit downregulation of the signal transducer and activatorof transcription-1 (STAT-1), IL-33, ICAM-1 and chemokine TARC/CCL17 following treatment with isosecotanapartholide obtained from *Artemisia princeps* [95].

## 7. The Effect of Plant Secondary Metabolite Treatment on Keratinocytes Exposed to UV-Radiation

UV radiation is an environmental-damaging agent that causes oxidative stress, resulting in damage to cellular components and apoptosis. It is also known to induce inflammatory conditions that lead to skin disorders. 

Keratinocytes are the first line of defense against UV radiation [96]. Exposure to UVB has been associated with changes in gene expression in epidermal cells [97]. A study of NHEKs found changes in expression in 249 of 539 studied genes four hours after exposure and in all 539 genes 24 h after exposure. The early cell response is mainly related to transcriptional arrest; however, the late cell response is more complex [98].

Following exposure to UV radiation (UVA, UVB and UVC mixed), keratinocytes prepared from newborn CD-1 mouse skin demonstrated EGFR receptor activation by phosphorylation of Tyr-992/Tyr-1045/Tyr-1068/Tyr-1173 residues via mechanisms associated with ROS. The EGFR receptor is strongly mitogenic and directly regulates the response of keratinocytes to UV radiation [99]. This activation may result in the induction of MAPK, phosphoinositide 3-kinase (PI3K)/Akt and NFκB signal transduction pathways [100]. These cascades regulate cell proliferation, differentiation and death [27,101,102]. It has been found that *Egfr* knockout mice are able to stay alive for only a few days after birth [99].

UVA and UVB exposure induces different groups of factors. UVB exposure induces the activation of c-Jun amino-terminal kinases (JNK), a member of the MAPKs in NHEKs [103], as well as JNK1 and JNK2 Th-183/Tyr-185 phosphorylation followed by the increased c-Fos expression [104]. It has also been found to induce transcription factor AP1 via the JNK pathway [105], and to induce Ser-473 phosphorylation of Akt [104] and Ser-727 phosphorylation of STAT3, known to play crucial roles in cell division, survival as well as migration [106]. UVA exposure of NHEKs cells induces Th-202/Tyr-424 phosphorylation of ERK1 and ERK2 and Th-180/Tyr-240 phosphorylation of p38, another member of the MAPKs family, followed by c-Jun overexpression. Moreover, mTOR is phosphorylated at Thr-2448 and p70S6k at Thr-421/Ser-424, both being downstream targets of Akt, and PI3K expression was found to be upregulated (p85), this being an upstream target of Akt. STAT 3 is phosphorylated at Tyr-705. In addition, nuclear translocation of NFκB proteins appears to be responsible for the induction of apoptosis [104].

NHEKs and immortalized human HaCaT keratinocytes also exhibit MAPKs signaling activation following exposure to both UVA and UVB radiation. The HaCaT cells demonstrate two-fold or even four-fold higher activity of p38 and JNK than NHEK, but the latter release more TNFα. The data also indicate that immortalized cells do not demonstrate NFκB pathway induction [107]. 

HaCaT keratinocytes exposed to both UVA and UVB radiation exhibit different gene expression profiles depending on a single or repetitive dose. Exposure to a single dose was associated with upregulation of the G/S checkpoint of the cell cycle, as well as NFκB, IL-1, death receptor and p38 signaling and downregulation of genes that regulate G2/M checkpoint of cell cycle and ATM serine/threonine kinase signaling. Additionally, numerous regulators including forkhead box M1 (FOXM1), forkhead box O1 (FOXO1), activating transcription factor 4 (ATF4), ATF6, melanocyte inducing transcription factor (MITF) and ETS homologous factor (EHF) were inhibited, while lysine demethylase 5B (KDM5B), SMAD family member 4 (SMAD4), tumor protein p63 (TP63), nuclear protein 1, transcriptional regulator (NUPR1), cyclin-dependent kinase inhibitor 2A (CDKN2A), NOTCH11 and STAT3 were activated. After repeated doses, interferon signaling was downregulated. Specific regulators such as FOXM1, TP73, NFE2 like bZIP transcription factor 2 (NFE2L2), MITF and EHF were inhibited, whereas STAT3, NUPR1, chromobox 5 (CBX5), ATF3, SMAD4 and MYC proto-oncogene, bHLH transcription factor (MYC), were activated [108]. In addition, HaCaT cells demonstrate strong activation of JNK but weak activation of ERKs following exposure to UVB [109].

Exposure to UV radiation also causes collagen destruction. The enzymes responsible for such effects are named matrix metalloproteinases (MMPs). In vivo data indicate that UV irradiation induces the expression of MMP-1, MMP-3 and MMP-9 mainly in the human epidermis [110]. Keratinocytes exposed to UVB exhibit accelerated MMP-1 expression, which may be mediated by the PKC-dependent induction of transient receptor potential vanilloid type 1 (TRPV1) followed by Ca2+-influx. This receptor is believed to be activated by various stimuli including capsaicin, heat or acids [111]. Moreover, keratinocytes treated with UVB exhibit elevated levels of MMP-1. These levels are driven by cascades dependent on ROS and ERK activation of the BLT2 receptor for leukotriene B4 (LTB4) and 12(S)-hydroxyeicosatetraenoic acid (12(S)-HETE) [112]. Additionally, UVB-irradiated keratinocytes may mediate a signal to the fibroblasts and enhance MMP-1 production [113]. The induction of MMP-9 and involucrin production were also observed on cultured keratinocytes after UVB treatment [114].

Human keratinocytes exposed to UV radiation result in ROS accumulation, as well as apoptosis by the intrinsic and extrinsic pathways [115]. Induction of programmed cell death is an important mechanism that may provide protection against skin cancer [116]. ROS generation and DNA damage induce p53 protein activation and cell cycle arrest followed by DNA repair or apoptosis [117]. TP53 knockout mice exhibit lower amounts of sunburn cells in the epidermis after exposure to UV radiation in comparison to wild-type mice [118]. In NHEKs, elevated TP53 expression after irradiation is related to phosphorylation of Ser-9/Ser15 followed by Apaf-1 and caspase-3 activation [119]. It also has been shown that p53 can interact with Bcl-2 and Bcl-xL proteins and regulate the intrinsic apoptosis pathway [120]. However, it has been found that combined UVB and heat treatment of NHEKs causes DNA damage but reduces the events of apoptosis in comparison to UVB alone, probably by the downregulation of the p-53-mediated response [121]. However, the process of apoptosis in basal keratinocytes exposed to UV radiation may also be independent of p-53 protein activity [115]. Studies suggest that the basal keratinocyte layer repairs DNA damage or undergoes apoptosis more rapidly than the suprabasal layer [119].

UV-induced HaCaT cells express the TNF-family receptor CD95 and may undergo apoptosis via the extrinsic pathway [122]. The data also show that UVB radiation induces caspase 8 in NHEKs and promotes the extrinsic pathway, as well as suppresses Bcl-2 and promotes the intrinsic pathway [123]. Similar results were obtained for HaCaT cells exposed to UVB radiation, in which both intrinsic and extrinsic apoptosis was induced [124]. In NHEKs irradiated with UVB, caspase activation occurs in the following order: caspase 3, caspase 9 then caspase 8 [125]. UVB irradiated HaCaT cells overexpressing Bcl-2 prevent ROS release and procaspase 3 and procaspase 8 activation [126]. The activation of caspases in UV-exposed NHEKs depends on protein kinase C (PKC). Caspase-3 and caspase-8 activation is prevented in blocked PKC [127].

Plant-derived compounds may counteract the detrimental effects of UV exposure on human skin. Data indicate that oil in water (O/W) emulsion containing 5% (*w*/*w*) Euterpe oleracea extract demonstrated a PF-UVA value (i.e., protection against UVA) of about 15 and a ratio of SPF (i.e., protection against UVB) to PF-UVA of about 1.5 [128]. Other extracts demonstrating great potential against UV radiation were obtained from *Helichrysum arenarium*, Sambucus nigra and *Crataegus monogyna*. All of them exhibit SPF values above 6 when used alone in O/W emulsion at 10% (wt.%), and above 16.5 and PF-UVA above 7.5 when used in combination [129]. *Hylocereus polyrhizus* at 1 mg/mL demonstrated an SPF of about 35 [130]. *Pterodon emarginatus* extract at 10% (*w*/*w*) in three different formulations (Lanette^®^, Polawax^®^ and Focus Gel^®^) exhibited an SPF value of about 8 [131]. Furthermore, 1% (*w*/*w*) extract of *Olea europaea* standardized to 20% oleuropein exhibited an SPF value of about 21 used in the special formulation [132]. A methanolic extract of *Washingtonia filifera* exhibits an SPF value of about 3.4 [133]. A phenolic compound-enriched fraction of *Salicornia ramosissima* extract at 10% (*w*/*w*) was found to have both SPF and PF-UVA values of about 13.5 when applied as O/W emulsion [134], whereas a flavonoid-enriched fraction of the *Vitis vinifera* extract at a concentration of 250 μg/mL had an SPF of about 18.6 and a PF-UVA of about 3.2 [135].

The most important group of compounds that may be used as sunscreen agents are phenolic compounds. These molecules equipped with aromatic rings are able to absorb UVA and UVB radiation at wavelengths of 200–400 nm and scavenge ROS and modulate signaling processes. Two flavonoids, rutin and quercitin, provide SPF values of about 12 when used in 10% (*w*/*w*) O/W emulsion, whereas PF-UVA provides a value of about 14.5 [136]. Combining 0.1% (*w*/*w*) rutin with 6% benzophenone, a synthetic organic filter, increased the SPF value from about 24 to 33 [137]. Similarly, synergistic effects were observed between 0.1% rutin (*w*/*w*), 1% benzophenone (*w*/*w*) and 3.5% (*w*/*w*) ethylhexyl methoxycinnamate [138]. Another promising sunscreen agent is named silymarin: a 50 µmol/L ethanol solution of silymarin yielded an SPF value of about 5.5, and its main constituent silybin provided a value of 6.0. PF-UVA was about 1.5 for both [139]. 

Choquenet tested twelve phenolic compounds at various concentrations, including myricetin, luteolin, apigenin, puerarin, baicalin, baicalein, hesperidin, hesperetin, naringenin, diosmin, caffeic acid and chlorogenic acid. Of these, chlorogenic acid and apigenin were found to be the best UVB and UVA filters, with SPF values at about 10 and 7 and PF-UVA values at about 9 and 6, respectively [140]. Similarly, among fifteen tested phenolic compounds at 7% (*w*/*v*) concentrations (resveratrol, piceid, catechin, quercetin, kaempferol, galangin, apigenin, naringenin, chrysin, pinocembrin, coumaric acid, ferulic acid, caffeic acid, caffeic acid phenylethyl ester and dimethyl caffeic acid), the highest SPF value was observed for apigenin, i.e., about 29 [141]. 

The plant extracts and single compounds may also modulate the UV-radiated keratinocytes response via different mechanisms. Both may downregulate the ROS level and upregulate the antioxidative enzyme level in keratinocytes exposed to UV. Typically, the nuclear factor erythroid 2–related factor 2 (Nrf-2) pathway is induced, with NF-κB, p53, MAPK and Akt signaling being reduced. In addition, MMPs and prooxidative cytokine expression are suppressed. Table 3 presents the impact of selected plant extracts on UV-irradiated human keratinocytes. The presented phytochemicals clearly exhibit significant antioxidant and anti-inflammatory properties.

Mice treated orally with a red raspberry extract prior to exposure to UVB irradiation demonstrate less skin wrinkling, water loss and epidermal thickening in comparison to those that were not treated [143]. *Rhus javanica* extract suppresses COX-2 expression in mice exposed to UVB and has anti-wrinkle effects [151]. In addition, *Rosa multiflora* extract reduces TNF-α and MMP-13 production [154], and *Paeonia × suffruticosa* extract reduces MMP-1 production in mice exposed to UVB [160]. *Moringa oleifera* extract is found to protect against UVB-induced oxidative stress injuries in the epidermis of the mouse in vivo. PPARα induction is involved in the protective effect of the extract [39].

Studies have examined the protective roles of single compounds on epidermal cells, including 3,5-dicaffeoyl-epiquinic acid obtained from *Atriplex gmelinii* [167], quercetin 3,7-dimethyl ether 40-glucoside from *Nymphoides indica* [163], youngiasides A and C from *Youngia denticulatum* [146], ixerisoside A from *Ixeris dentata* [168], α-, β-, γ-mangostins and gartanin from *Garcinia mangostana* [169]. Of these, 3,5-dicaffeoyl-epiquinic acid downregulates the expression of MMP-1, -2 and -9, whereas α-, β-, γ-mangostins and gartanin downregulate the expression of MMP-1 and -9. In addition to these, youngiasides A and C also increased SOD1, Nrf2 and heme oxygenase-1 (HO-1) expression and downregulated MAPK and NF-κB pathways. Quercetin 3,7-dimethyl ether 40-glucoside also suppressed NF-κB as well as TNF-α, IL-1β, IL-6, IL-8, myelodysplastic syndromes (MDS) and thymus and activation-regulated chemokine (TARC). Ixerisoside A also blocked pro-inflammatory cytokines including IL-6 and IL-8, inhibited COX-2 expression and downregulated MAPKs. Keratinocytes exposed to UVA irradiation and then treated with prenylated phenols from *Artocarpus communis* exhibited reduced cell damage in contrast to untreated ones [170].

## 8. Modulatory Effect of Plant Secondary Metabolites on Keratinocytes Involved in the Wound Healing Process Triggered by Disruption of the Epidermal Barrier

Wound healing occurs as a consequence of skin barrier disruption. It is a complex process consisting of a series of phases, including hemostasis, inflammation, proliferation and remodeling [171]. The first phase is related to the formation of blood clots that prevent blood loss. The next phases are connected with the recruitment of numerous cells, including keratinocytes, to the wound site [172]. During inflammation, the debris is removed by neutrophils and macrophages. The proliferation phase is characterized by re-epithelialization and granulation performed by a mixture of stem cells, endothelial cells and keratinocytes. Both the inflammation and proliferation phases are related to new blood vessel formation. Finally, the wound healing is completed by the remodeling stage, in which the collagen matrix is restructured by fibroblasts [173].

Keratinocytes migrate, proliferate, and cross-talk with fibroblasts following wound contraction [174]. Activated keratinocytes are characterized by cytoskeleton changes, downregulation of K1 and K10 expression and upregulation of K6, K16 and K17 keratin expression, enabling them to migrate and restore the epidermal barrier [175,176]. The migratory rate is closely related with the disruption of adhesion between cells (desmosomes) as well as between cells and substratum (hemidesmosomes). 

Desmosomes are structures that are essential for cell–cell adhesion and skin integrity. The core desmosome protein is named desmoplakin. Desmosomal adhesion in response to wounding is regulated by PKCα [177]. It has been suggested that the transcription factor Slug is related to effective re-epithelialization and desmosomal disruption [178]. In addition, retinitis pigmentosa GTPase regulator interacting protein 1-like (RPGRIP1L) is believed to be an epithelialization factor, and Rpgrip1l knockout mice exhibit impaired desmosomal structure [179]. Problems with desmosome function may also be associated with loss of desmoplakin [180] and flotillin expression [181], as well as cadherin disruption [182]. Desmoplakin is essential for cadherin cluster formation [183], which is crucial for desmosome formation, as well as intercellular adhesion. In turn, cluster formation is regulated by flotillins [184].

Hemidesmosomes are structures that link cells into the basal layer and are mediated by integrins [185]. Integrins are considered as regulators of growth factor receptor pathways, and their activity is believed to elevate growth factor activity [186]. One integrin expressed by keratinocytes, α6β4, binds to laminin-5. α6β4 knockout mice demonstrate an absence of hemidesmosomes [187]. The modulation of α6β4 binding affinities is involved in keratinocyte motility [188]. In cultured keratinocytes, PKC or PKA stimulate hemidesmosome turnover by integrin β4 subunit phosphorylation [189]. In turn, this phosphorylation is modulated by the presence of EGF and macrophage-stimulating proteins in the wound [190]. 

Numerous other regulators take part in the process of keratinocyte migration and proliferation, including various growth factors from the tyrosine kinase, insulin, FGF, vascular endothelial growth factor and scatter factor families [191]. In addition, cytokines, chemokines, MMPs and extracellular macromolecules also play various roles [192]. 

The epidermal growth factor family of receptor tyrosine kinases (HER) are also known to play a role in keratinocyte activity. Keratinocytes express 1, 2 and 3 HER receptors. Their ligand, including epidermal growth factor (EGF), heparin-binding EGF like growth factor (HB-EGF) and TGF-α stimulate keratinocyte migration [191] and proliferation [193]. EGF agonists have been found to completely prevent keratinocyte migration [194].

Moreover, insulin secreted by β-cells in the pancreas is able to circulate into the skin, where it can then bind to insulin receptors (IR) expressed in skin keratinocytes. Insulin mediates keratinocyte migration and proliferation. A combination of insulin and TGF-α was found to act synergistically in this regard. An in vivo study in a mouse model found that topical application of insulin results in accelerated wound healing [195]. A similar effect was observed in a diabetic mouse model [196].

Additionally, keratinocyte insulin-like growth factor 1 (IGF-1) receptor and its ligand, IGF-1, synthesized by fibroblasts or hepatocytes, has been found to stimulate the motogenic effect in keratinocytes. That signaling is downregulated in diabetic wounds. IGF-1 and EGF act synergistically; the first by induction of the PI3K pathway, the second by the MAPK/ERK pathway [197]. IGF-1 also acts synergistically with HB-EGF in stimulating keratinocyte proliferation [198].

Fibroblast growth factor receptor 1 (FGFR1) and FGFR2 have been shown to stimulate keratinocyte migration and proliferation following binding by several ligands, including fibroblast growth factor 1 (FGF-1), -2, -7, -10 and FGF-1 -10, respectively. FGFR1 and FGFR2 knockout keratinocytes have reduced the motility that may result from a lack of expression of focal adhesion components due to the absence of an FGFR signal transduction pathway [199]. FGF-7 is highly expressed at the beginning of the wound healing process. This factor probably acts by compensatory or overlapping mechanisms because inhibiting their expression does not directly influence the overall rate of the process. FGF-7 knockout diabetic mice exhibit delayed wound healing [200]. FGF-10 with dermatan sulfate synergistically increased keratinocyte migration in a wound [201]. 

Keratinocytes also express VEGFR-1, -2, and -3. Their ligand, VEGF-A, is produced by keratinocytes and macrophages. In addition to granulation tissue formation and angiogenesis, that factor also promotes keratinocyte migration [202]. 

Keratinocytes also express the MET receptor tyrosine kinase (RTK). Their ligand, hepatocyte growth factor (HGF), is produced by fibroblasts and by keratinocytes after skin injury. Throughout the stimulation of STAT3 signaling, HGF induces keratinocyte motility. In addition, HGF activates VEGF-A expression and influences cell proliferation. The RON RTK receptor expressed by keratinocytes is activated by a macrophage-stimulating protein (MSP) produced by hepatocytes. RON activates the PI3K/Akt pathway; it also induces phosphorylation of both RON and integrin α6β4 at 14-3-3 binding sites, allowing the formation of RON and α6β4 complex by 14-3-3. Following this, α6β4 is relocated to the lamellipodia from hemidesmosomes. The induction of α3β1 is associated with keratinocyte spread on laminin-5, as well as activation of p38 and NF-κB signaling, which is required for cell migration [192]. 

Keratinocyte proliferation is also stimulated by granulocyte macrophage-colony stimulating factor (GM-CSF) produced by keratinocytes, among others. Following binding to the CD116 receptor, GM-CSF activates the JAK/STAT signaling pathway. During wound healing, GM-CSF plays an indirect role by the induction of secondary cytokines. Studies of transgenic mice overexpressing the GM-SCF factor indicate that keratinocyte-derived GM-CSF overexpression takes place in the basal layer and is related to accelerated wound contracting. In the first phase of that process, significant mitogenic keratinocyte growth and granulation tissue formation were observed, as well as different regulation of TGF-β, IFN-γ, and iIL-6 release [203]. 

Another factor, angiopoietin-related growth factor (AGF), is able to bind to keratinocytes αv integrin via RGD-motif. Transgenic mice overexpressing AGF revealed increased proliferation of keratinocytes and more rapid wound closure compared to wild-type controls. AGF is also produced by platelets and mast cells; however, AGF release takes place only on wounded skin. AGF is profusely expressed by hepatocytes [204].

High mobility group protein β1 (HMGB1) enhances keratinocyte migration by an unknown receptor. That factor is released by macrophages and monocytes. HaCaT keratinocytes exposed to HMGB1 exhibit accelerated migration and proliferation, as well as ERK1/2 pathway induction [205]. An in vivo study found that diabetic mice demonstrate lower levels of HMGB1 than non-diabetic mice. In addition, topical application of HMGB1 resulted in accelerated wound healing in diabetic mice, but not in normoglycemic mice. Moreover, it was observed that HMGB1 plays a chemotactic role on keratinocytes in vitro [206].

Hypoxia-inducible factor-1 (HIF-1), a regulator of oxygen homeostasis, is released during the hypoxia accompanying wound healing. The presence of HIF-1 results in elevated expression of heat shock protein 90 (HSP90) by keratinocytes and binding to LDL receptor-related protein 1 (LRP-1) followed by an increase in cell migration. HSP90 regulates the initial phase of wound healing [207,208] and acts synergistically with TGF-α [209]. An in vitro heat shock assay performed with HaCatT cells increases keratinocyte motility, whereas an in vivo assay performed on mice with thermal burns shows that topical application of HSP90 increases tissue granulation and reduces inflammation [210].

Cytokines including IL-1, IL-6 and TNF-α also regulate keratinocyte migration. IL-1 promotes the secretion of FGF-7 [211], while IL-6 stimulates motility via the STAT-3 dependent pathway [212]. 

Chemokines produced by various types of cells may also propagate keratinocyte motility. The N terminus takes part in receptor activation. Keratinocytes express CXCL1, 8, 10, 11 and CCR14, 17, 27, which stimulate their migration, whereas CXCL1, 8 and 12 accelerate their proliferation. Reduced keratinocyte motility and proliferation has been observed in CXCR2 knockout mice. CXCR2 is a receptor for CXCL1 (growth-related oncogene-α) and CXCL8 (IL-8) chemokines, which are essential for keratinocyte activity [208]. CXCL10 and CXCL11 are expressed by basal keratinocytes during the re-epithelialization phase [213]. CXCR3 is a receptor for CXCL10 and CXCL11. CXCR3 knockout mice demonstrate delayed re-epithelization [214]. Interestingly, keratinocyte migration and wound closure were accelerated in mice with thick wounds in the presence of CXCL11 [215]. Conversely, reduced proliferation was observed in keratinocytes exposed to an elevated level of CXCL8 [216].

Neuropeptides, molecules released, among others, by sensory neurons during cutaneous injury, can also accelerate keratinocyte motility and proliferation [217]. The G protein-coupled receptor (GCRP) increases the mitogenic potential of keratinocytes [218]. Vasoactive intestinal peptide (VIP) was found to directly induce keratinocyte migration in vitro [219], or by the upregulation of the production of TGF-α [220] and VEGF [221]. Substance P (SP), which binds to the neurokinin 1 receptor (NK1R), and calcitonin gene-related peptide (CGRP) stimulate the production of inflammatory agents including IL-1, IL-6, TNF-α and nerve growth factor (NGF) in keratinocytes, as well as their motility. Similarly, SP and NK1R have been found to promote human and murine keratinocyte proliferation [222,223,224,225,226]. SP and NK1R SP activate all three members of the MAPK family, whereas CGRP only induces p38 and ERK1/2 [227]. Keratinocytes under hypoxia and poor nutrient environments similar to chronic wounds exhibit accelerated cell proliferation when exposed to SP, and mice with full-thickness wounds demonstrated elevated wound closure after topical application of SP [228]. Diabetic wounded mice also exhibited accelerated re-epithelialization after SP exposure [229]; however, an SP damage agent named neutral endopeptidase (NEP) was also found to be upregulated in diabetic mice wounds. This may be due to the impaired activities of keratinocytes under the influence of NEP inhibitors [230]. It was observed that, under hyperglycemic conditions, keratinocytes demonstrate lower expression of the neuropeptide neurotensin (NT) and neurotensin receptor (NTR); however, the addition of exogenous NT do not impact keratinocyte proliferation but even reduced migration [231]. An in vitro study on keratinocytes indicated increased expression of IL-1α, IL-8, TNF-α and NGF mRNA following treatment with SP, CGRP, VIP and galanin (GAL) [232]. It was also found that keratinocyte expresses the GAL receptor GALR2, which is known to influence keratinocyte proliferation [233]. In addition, β-endorphin also appears to enhance keratinocyte motility by binding with mu-opiate receptor [234].

Keratinocytes also express cholinergic receptors, including nicotinic (nAChR), including α3, α5, α7, α9, α10, β1, β2, and β4 and muscarinic acetylcholine (ACh) (mAChR) forms, including M1, 2, 3, 4, and 5. Increased keratinocyte migration is observed after the activation of the following receptors: α3 by PKCδ and RhoA signaling; α7 by PI3K and Rac/Cdc42 signaling; α9 by modulating adhesion between cells and between the cell and extracellular matrix (ECM) and M4 by integrins α5β1, αvβ5 and αvβ6. Conversely, M3 activation upregulates integrins α2β1 and α3β1, related to adhesion, and suppresses keratinocyte migration [235,236,237]. 

In wounds, keratinocyte migration onto the dermal matrix and MMP-1 expression is mediated by native type I collagen [238]. In vitro data indicate that MMP-1 fragmented the collagen, which was followed by impaired keratinocyte function and slow spread [239]. Interestingly, it has been suggested that chronic wounds, characterized by accelerated IL-1β and TNF-α expression, result in elevated levels of MMPs and the secretion of growth factors such as FGFs. This results in reduced keratinocyte migration due to degradation of ECM components and the greater availability of selected growth factors [172]. Throughout proteolytic activity, MMPs convert the latent form of growth factors into active ones, including IGF-1 [240].

The plant extracts and their component compounds may modulate keratinocyte migration and proliferation rates via various mechanisms, including stimulation of the MAPK and PI3K/AKT signal transduction pathways. In some studies, a higher expression of β1-, α6-, β4-integrin and E-cadherin were observed after extract treatment. Table 4 presents the impact of selected plant extracts on the migration and proliferation rates of human keratinocytes. As cellular motility is closely connected with the induction of various growth factors, the presented plant-derived compounds exhibit important properties that modulate the production of growth factors and enhance migration followed by accelerated wound closure.

A study of the abovementioned *Aegle marmelos* [253], *Boerhavia diffusa* [254] and *Stellera chamaejasme* [260] extracts found them to reduce wound area in rats. Annona reticulata [255] and *Centella asiatica* [259] also demonstrated acceleration of wound healing after topical application in mice and rabbits, respectively. 

Finally, cyanidin-3-glucoside derived from blackberry [263] and chlorogenic acid derived from *Parrotia persica* [264] significantly accelerate the wound closure process in keratinocytes in vitro.

## 9. Conclusions

Plants are key sources of secondary metabolites that exert various antioxidant and anti-inflammatory effects, among others. These compounds are able to modulate signaling pathways in numerous cells, including epidermal cells. Keratinocytes placed in the outer layer of the skin create a physical barrier against harmful stimuli. They are particularly vulnerable to UV-radiation, related oxidative stress and inflammation. Any disruption of the epidermis stimulates cell migration, proliferation and participation in wound healing. It is suggested that such compounds of plant origin may be used to modulate keratinocyte function by improving ROS scavenging, inhibiting inflammation and accelerating wound healing via influencing signal transduction pathways. Hence, plant extracts and their component compounds may have an impact on keratinocyte biology and its ability to maintain homeostasis. However, further in vitro and in vivo studies of the mechanisms of the action of phytochemicals, and more specific toxicity and clinical studies are needed to ensure the effectiveness and safety of plant compounds for use on human skin.

## Figures and Tables

**Table 1:** Selected plant extracts from different species with identified compounds and their in vitro effect on H_2_O_2_-stimulated human keratinocytes.

Name of the Species/Family	Part of the Plant	Type of Extract	Cell Line	Identified Compounds	Mechanism of Action	Effects	Ref.
*Andrographis paniculata* (Burm.f.) Nees (Acanthaceae)	leaves	methanolic	HaCaT stimulated by hydrogen peroxide	andrographolide	Inhibition: ROS production	antioxidant	[37]
*Acer rubrum* L. (Sapindaceae)	leaves	Maplifa^TM^	HaCaT stimulated by hydrogen peroxide and methylglyoxal	ginnalin A	Induction: cell viability Inhibition: ROS production, caspases-3/7 and -8 release	antioxidant cytoprotective anti-apoptotic	[38]
*Moringa oleifera* Lam. (Moringaceae)	stem	ethanolic	HaCaT stimulated by hydrogen peroxide	luteolin, rutin, quercetin	Induction: SOD and CAT, activation of PPARα Inhibition: ROS production	antioxidant	[39]
*Lagerstroemia speciosa* (L.) Pers. and *Lagerstroemia floribunda* Jack (Lythraceae)	flowers	ethanolic	HaCaT stimulated by hydrogen peroxide	ellagic acid, epicatechin gallate, and quercetin	Inhibition: hydrogen peroxide-induced cell death	antioxidant anti-apoptotic	[40]
*Punica granatum* L. (Lythraceae)	fruits	Pomella^®^	HaCaT stimulated by hydrogen peroxide	punicalagins, ellagic acid and urolithin A	Inhibition: ROS production, apoptotic cells formation, caspase 3 production	anti-oxidant anti-apoptotic	[41]
*Castanea sativa* Mill. (Fagaceae)	chestnut shells, and inner chestnut shells	aqueous	HaCaT stimulated by hydrogen peroxide	gallic acid	Inhibition: oxidized lipids, NO and iNOS production, collagen degradation	cytoprotective antioxidant	[42]
*Oenothera biennis* L. (Onagraceae)	aerial parts	ethanolic	HaCaT stimulated by hydrogen peroxide	3-caeoylquinic acid, ellagic acid, and quercetin 3-O-glucuronide, quercetin	Induction: cell viability, heme oxygenase-1 (HO-1) Inhibition: DNA damages, caspase-3, PARP	cytoprotective antioxidant anti-apoptotic	[43]
*Himantoglossum robertianum* (Loisel.) P.Delforge (Orchidaceae)	flowers	ethanolic	HaCaT stimulated by hydrogen peroxide	flavones and flavan-3-ols, scopoletin, and phenolic acids	Induction: cell viability and motility Inhibition: elastase and collagenase	cytoprotective antioxidant stimulate migration	[44]
*Myrciaria dubia* (Kunth) McVaugh (Myrtaceae)	fruit	ethanolic	HaCaT stimulated by high glucose	ellagic acid and quercetin	Induction: Nrf2 Inhibition: MAPK/AP-1, NF-κB	antioxidant anti-inflammatory	[45]
*Clitoria ternatea* L. (Fabaceae)	flowers	aqueous	HaCaT stimulated by hydrogen peroxide	anthocyanins derived from delphinidin, including polyacylated ternatins, and flavonol glycosides derived from quercetin and kaempferol	Inhibition: cytotoxicity effects of H_2_O_2_	antioxidant	[46]

**Table 2:** Selected plant extracts from different species with identified compounds and their in vitro effect on TNF-α or IFN-γ-stimulated human keratinocytes.

Name of the Species/Family	Part of the Plant	Type of Extract	Cell Line	Identified Compounds	Mechanism of Action	Effects	Ref.
*Rydingia persica* (Burm.f.) Scheen & V.A.Albert (Lamiaceae)	aerial parts	methanolic	HaCaT stimulated by LPS	labdane-type diterpenoids	Inhibition: IL-6 and TNF-α release	anti-inflammatory	[78]
*Andrographis paniculata* (Burm.f.) Nees (Acanthaceae)	leaves	methanolic	HaCaT stimulated by LPS/TNF-α	andrographolide	Induction: IL-8 secretion Inhibition: TNF-α expression	anti-inflammatory	[37]
*Curcuma aromatica* Salisb. (Zingiberaceae)	rhizome	ethanolic	HaCaT stimulated by TNF-α	germacrone, curdione, dehydrocurdione, zederone, curcumenol, curcumin	Inhibition: NF-κB activation	anti-inflammatory	[79]
*Crateva adansonii* DC. (Capparaceae)	leaves	aqueous	HPEKs infected by Staphylococcus aureus	quercitrin, isoquercitrin, quercetin-3-O-(b-d-xylopyranosyl-a-l-rhamnopyranoside)	Inhibition: IL-6, IL-8 and TNFα expression	anti-inflammatory	[80]
*Perilla frutescens var. crispa* (Thunb.) H.Deane (Lamiaceae)	leaves	ethanolic	HaCaT stimulated by TNF-α/IFN-γ	caffeic acid, rosmarinic acid, luteolin	Inhibition: p38, ERK, and JNK expression; STAT-1 and NK-κB activation	anti-inflammatory	[81]
*Rhus coriaria* L. (Anacardiaceae)	fruits	ethanolic	HaCaT stimulated by TNF-α	rutin, quercetin derivative, gallotannins	Inhibition: NF-κB activation; ICAM-1, and MMP-9 secretion	anti-inflammatory	[82]
*Ampelopsis glandulosa* (Wall.) Momiy. (Vitaceae)	rhizome	ethanolic	HaCaT stimulated by TNF-α/IFN-γ	betulin, betulinic acid, β-sitosterol, β-5 sitosterol glucoside, dihydrokaempferol, dihydrokaempferol 3-O-glycoside, catechin, gallic acid, vanillic acid, ethyl gallate, ethyl gallate 4-O-β-D7glucopyranoside, syringic acid, benzyl 6ʹ-O-galloyl-β-d-glucopyranoside, ellagic acid, 3ʹ-O-methylellagic acid 4-O-α-l-rhamnopyranoside, 3,3′4′-O-tri-methylellagic acid 4-O-β-d-glucopyranoside, and resveratrol	Inhibition: TNF-α, IL-6, IL-1β, and CCL17 expression; STAT-1, NK-κB, ERK and p38 activation	anti-inflammatory	[83]
*Sanguisorba officinalis* L. (Rosaceae)	roots	ethanolic	HaCaT stimulated by TNF-α/IFN-γ	(+)-catechin, (–)-epicatechin, ziyuglycoside	Inhibition: macrophage-derived chemokine (MDC), normal T-cell expressed and secreted (RANTES), IL-8 and thymus and activation regulated chemokine (TARC) production; STAT-1, ERK and NF-κB activation	anti-inflammatory	[84]
*Gleditsia sinensis* Lam. (Fabaceae)	thorns	ethanolic	HaCaT stimulated by TNF-α/IFN-γ	(+)catechin, epicatechin, eriodictyol and quercetin, caffeic acid and ethyl gallate	Inhibition: MDC and TARC production	anti-inflammatory	[85]
*Morus alba* L. (Moraceae)	barks	aqueous	HaCaT stimulated by TRAIL	moracin O and P	Induction: antiapoptotic proteins Bcl-xL and Bcl-2 Inhibition: NFκB activation	anti-inflammatory anti-apoptotic	[86]
*Morus alba* L. (Moraceae)	root bark	ethanolic	HaCaT stimulated by TNF- α/IFN-γ	kuwanon G and morusin	Inhibition: RANTES/CCL5, TARC/CCL17, and MDC/CCL22 secretion; STAT 1 and NF-κB activation	anti-inflammatory	[87]
*Combretum collinum* Fresen. (Combretaceae)	leaves	aqueous	HaCaT stimulated by TNF-α	myricetin-3-O-rhamnoside and myricetin-3-O-glucoside	Inhibition: IL-8 secretion	anti-inflammatory	[88]
Aucklandia lappa DC. (Asteraceae)	whole extract	methanolic	HaCaT stimulated by TNF-α/IFN-γ	alantolactone, caryophyllene, costic acid, costunolide, and dehydrocostuslactone	Inhibition: TARC, RANTES, MDC and IL-8 production; STAT1 activation	anti-inflammatory	[89]
*Quercus mongolica* Fisch. ex Ledeb. (Fagaceae)	leaves	acetone	HaCaT stimulated by LPS	pedunculagin	Inhibition: IL-6 and IL-8 production	anti-inflammatory	[90]
*Melaleuca styphelioides* Sm. (Myrtaceae)	leaves	methanolic	NCTC 2544 keratinocytes stimulated by IFN-γ/histamine	quercetin, gallic acid, ellagic acid	Inhibition: ICAM-1, iNOS, COX-2, NF-κB	anti-inflammatory antioxidant	[91]
*Carpinus tschonoskii* Maxim. (Betulaceae)	leaves	ethanolic	HaCaT cells stimulated by LPS	tellimagrandin I	Inhibition: IL-6 production	anti-inflammatory	[92]

**Table 3:** Selected plant extracts from different species with identified compounds and their in vitro effect on UV-radiation-stimulated human keratinocytes.

Name of the Species/Family	Part of the Plant	Type of Extract	Cell Line	Identified Compounds	Mechanism of Action	Effects	Ref.
*Petasites japonicus* (Siebold & Zucc.) Maxim. (Asteraceae)	leaves	methanolic	NHEKs exposed to UVB irradiation	kaempferol-3-O-(6″-acetyl)-β-d-glucoside, quercetin-3-O-(6″-acetyl)-β-d-glucoside, kaempferol-3-O-β-d-glucoside, and quercetin-3-O-β-d-glucoside	Induction: Nrf2 and heat-shock response transcription elements (HSE) that resulted in the induction of heme oxygenase-1 (HO-1) and HSP70, respectively	Protection against UV-induced cell damages, anti-apoptotic	[142]
*Rubus idaeus* L. (Rosaceae)	fruits	ethanolic	HaCaT exposed to UVB radiation	cyanidin, ellagic acid, pelagonidin-3-sophoroside, methylquercetin-pentose conjugate, and cyanidin-3-rutinoside	Induction: SOD, Nrf2, and HO-1. Inhibition: caspase-3, c-jun modulation; NF-κB and COX-2 activation	antioxidant, anti-apoptototic anti-inflammatory	[143]
*Castanea sativa* Mill. (Fagaceae)	leaves	methanolic	HaCaT exposed to UVB radiation	crenatin, chestanin, gallic acid, cretanin, 5-O-p-coumaroylquinic acid, p-methylgallic acid and quercetin-3-O-glucoside	Inhibition: p53 expression	protection against UVB-induced cell damages, antioxidant	[144]
*Potentilla kleiniana* Wight et Arn (Rosaceae)	whole plant	ethanolic	HaCaT exposed to UVB radiation	diosmetin-7-O-neohesperidoside, dimethylellagic acid hexose, zizybeoside I, 4-O-[b-d-xylopyranosyl]-3,30-di-O-methylellagic acid, and buddlenol A	Inhibition: caspase-3	cytoprotective effect	[145]
*Crepidiastrum denticulatum* (Houtt.) Pak & Kawano (Asteraceae)	whole plant	ethanolic	HaCaT exposed to UVB radiation	chicoric acid, 3,5dicaffeoylquinic acid, chlorogenic acid, luteolin 7-O-glucuronide, youngiaside A, youngiaside B, youngiaside C	Induction: antioxidant enzymes expression Inhibition: ROS release, MAPKs, AP-1 and NF-κB activation	antioxidant, anti-inflammatory	[146]
*Vitis vinifera* L. (Vitaceae)	leaves	aqueous	HaCaT exposed to UVB radiation	caftaric acid, rutin, hyperoside, quercetin 3-*O*-glucoside, quercetin 3-O-glucuronide, kaempferol 3-O-glucoside, delphinidin 3-O-glucoside, cyanidin 3-O-glucoside, petunidin 3-O-glucoside, peonidin 3-O-glucoside, malvidin 3-O-glucoside	Inhibition: IL-8 secretion	anti-inflammatory	[147]
*Dalbergia odorifera* T.C.Chen (Fabaceae)	heartwood	ethanolic	HPEKs exposed to UVB radiation	sativanone	Inhibition: ROS release, p53 and p21 protein production	antioxidant, anti-senescence	[148]
*Opuntia ficus-indica* (L.) Mill. (Cactaceae)	stems	aqueous	HaCaT exposed to UVA radiation	eucomic and piscidic acids	Inhibition: ROS production, lipid peroxidation and GSH depletion	antioxidant	[149]
*Melissa officinalis* L. (Lamiaceae)	leaves	ethanolic	HaCaT exposed to UVB radiation	rosmarinic acid, salvianolic acid derivatives, caffeic acid and luteolin glucuronide	Inhibition: ROS production, DNA damage and DNA damage response	cytoprotective antioxidant	[150]
*Rhus javanica* L. (Anacardiaceae)	whole plant	ethanolic	HaCaT exposed to UVB radiation	gallic acid, 5-O-galloyl-β-d-glucose, Methyl gallate, Syringic acid, Protocatechuic acid	Inhibition: COX-1, MMP-1 exprwssion; MAPK, AKT, EGFR activity	antioxidant, anti-inflammatory	[151]
*Juglans regia* L. (Juglandaceae)	flowers	methanolic	HaCaT exposed to UVB radiation	3,7-dimethyl-1,6-octadiene, pentadecanoic acid, 14-methyl, methyl ester, 2-(2,6-dimethoxy-benzoylamino)-propionic acid, ethyl ester, hexadecanoic acid, ethyl ester (palmitic acid), 10-octadecenoic acid, methyl ester, erucic acid; 1,2,3-benzothiadiazole; estra-1,3,5(10),6-tetraene-3,17-diol, (17β)-; 17-acetate, 2,2,4-trimethyl-3- (3,8,12,16-tetramethyl-heptadeca-3,7,11,15-tetraenyl)-cyclohexanol and oleic acid, trimethylsilyl ester	Inhibition: ROS production, lipid peroxidation, TNF-α, IL-1, IL-6, NF-κB, COX-2 activation	antioxidant, anti-inflammatory	[152]
*Portulaca oleracea* L. (Portulacaceae)	whole plant	methanolic	HaCaT exposed to UVB radiation	portulacanone A and portulacanon D	Induction: SOD expression, and HO-1 via Nrf2 pathway Inhibition: ROS production	antioxidant	[153]
*Rosa multiflora* Thunb. (Rosaceae)	flowers	ethanolic	HaCaT exposed to UVB radiation	quercitrin, hyperin, and isoquercetin	Inhibition: ROS production, IL-6, IL-8 MMP1; NF-κB activation	anti-oxidant anti-inflammatory	[154]
*Rhodomyrtus tomentosa* (Aiton) Hassk. (Myrtaceae)	fruits	ethanolic	NHEKs exposed to UVB radiation	piceatannol	Inhibition: cyclobutane pyrimidine dimers formation, prostaglandin E2 secretion Induction: enzyme activity of the DNA polymerases	cytoprotective anti-inflammatory	[155]
*Cecropia obtusa* Trécul (Urticaceae)	leaves	methanol	HaCaT exposed to UVB radiation	chlorogenic acid, luteolin-C-hexoside, luteolin-Chexose-O-deoxy-hexose, and apigenin-C-hexose-O-deoxy-hexose	Inhibition: MMP-1, IL-1β and IL-6	anti-inflammatory	[156]
* Scutellaria baicalensis * Georgi (Lamiaceae)	roots	ethanolic	HaCaT exposed to UVB radiation	baicalin, wogonoside, baicalein and wogonin	Induction: HO-1; Nrf2 activation Inhibition: MMP-1, IL-6; MAPK, AP-1 and NF-κB activation	cytoprotective anti-inflammatory antioxidant	[157]
*Spatholobus suberectus* Dunn (Fabaceae)	stem	aqueous and ethanolic	HaCaT exposed to UVB radiation	gallic acid, catechin, vanillic acid, syringic acid and epicatechin	Inhibition: ROS production; MAPKs, NF-κB, c-Jun activation	anti-inflammatory antioxidant	[158]
*Adenocaulon himalaicum* Edgew. (Asteraceae)	leaves	ethanol	HaCaT exposed to UVB radiation	neochlorogenic acid	Induction: filaggrin, involucrin, loricrin expression Inhibition: MMP-1; MAPK, AP-1 activation	hydration anti-inflammatory antioxidant	[159]
*Paeonia × suffruticosa* Andrews (Paeoniaceae)	roots	ethanol	HaCaT exposed to UVB radiation	paeonol	Induction HO-1; Nrf2 activation Inhibition: MAPK	cytoprotective, antioxidant	[160]
*Aquilaria crassna* Pierre ex Lecomte (Thymelaeaceae)	leaves	aqueous/ethanolic	NHEKs exposed to UVB radiation	iriflophenone 3,5-C-β-d-diglucoside, iriflophenone 3-C-β-d-glucoside, mangiferin and genkwanin 5-O-β-primevoside	Inhibition: IL-1α, IL-8 and prostaglandin E2 (PGE2)	anti-inflammatory	[161]
*Aloe vera* (L.) Burm.f. (Asphlodelaceae)	flowers	aqueous	HaCaT exposed to UVB radiation	isoorientin	Induction: involucrin expression	hydration	[162]
*Nymphoides indica* (L.) Kuntze (Menyanthaceae)	whole plant	methanolic	HaCaT exposed to UVB radiation	quercetin 3,7-dimethyl ether 4′-glucoside	Induction: filaggrin, involucrin, loricrin expression Inhibition: MAPK, NF-κB activation	hydration cytoprotective antioxidant	[163]
*Biancaea sappan* (L.) Tod. (Fabaceae)	whole plant	methanolic	NHDKs exposed to UVBA radiation	brazilin	Induction: glutathione peroxidase 7	antioxidant	[164]
*Clitoria ternatea* L. (Fabaceae)	flowers	aqueous	HaCaT exposed to UVB radiation	delphinidin, including polyacylated ternatins, and flavonol glycosides derived from quercetin and kaempferol	Inhibition: mtDNA damage	cytoprotective	[46]
*Syzygium formosum* (Wall.) Masam (Myrtaceae)	leaves	ethanolic	HaCaT exposed to UVB radiation	triterpenic acids	Inhibition: IL-1β, IL-6, IL-8 and COX-2 expression	anti-inflammatory	[165]
*Aster yomena* (Kitam.) Honda. (Astereae)	callus	aqueous	HaCaT exposed to UVB radiation	robustic acid, 3,5-Di-O-methyl-8-prenylafzelechin-4beta-ol, acetylpterosin C and pterosin N, L-thyronine, 3,4-dicaffeoyl-1,5-quinolactone, dehydrophytosphingosine and phytosphingosine, α-linolenic acid, palmitic amide, olemaide, and 13Z-docosenamide, and glycerophospholipids	Inducttion: type I procollagen synthesis, TGF-β expression Inhibition: ROS production, elastase production, MMP-1, MMP-3, MMP-9, TNF-α, IL-1β, IL-8 expression	cytoprotective antioxidant anti-inflammatory	[166]

**Table 4:** Selected plant extracts from different species with identified compounds and their in vitro effect on human keratinocyte migration and proliferation rates.

Name of the Species/Family	Part of the Plant	Type of Extract	Cell Line	Identified Compounds	Mechanism of Action	Effects	Ref.
*Boesenbergia rotunda* (L.) Mansf. (Zingiberaceae)	rhizomes	ethanolic	HaCaT	kaempferol	Induction: ERK 1/2, Akt Activation: MAPK and PI3K/Akt pathways	stimulate proliferation	[241]
*Rubus fruticosus* L. (Rosaceae)	leaves	aqueous	HaCaT	phenolic compounds	-	stimulate migration	[242]
*Alternanthera sessilis* (L.) R.Br. ex DC. (Amaranthaceae)	stems	ethanolic	HaCaT	2,4-dihydroxy-2,5-dimethyl-3(2H)-furan-3-one, hexadecanoic acid <n->, 2-1,2,4-trioxolane,3-phenyl-, palmitate <ethyl- and L-glutamic acid	-	stimulate migration and proliferation	[243]
*Hibiscus syriacus* L. (Malvaceae)	leaves	ethanolic	HaCaT	flavonoids, coumarins	-	stimulate migration	[244]
*Digitaria sanguinalis* (L.) Scop. (Poaceae)	flowers	ethanolic	HaCaT	xycaine, hexadecanoic acid, linolenic acid, octadecanoic acid, phenol, 2,2′-methylenebis [6-(1,1-dimethylethyl)-4-methyl-, pentacosane, heptacosane, squalene, 1-docosene, cyclooctacosane, campesterol, stigmasterol, lanosterol, multiflora-7,9(11)-dien-3β-ol, sitostenone	-	stimulate proliferation	[245]
*Fuchsia magellanica* Lam. (Onagraceae)	leaves	aqueous, ethanolic	HaCaT	gallic acid derivatives, hydroxycinnamic acid derivatives and flavonoid glycosides, anthocyanins	-	stimulate migration	[246]
*Elaeagnus umbellata* Thunb. (Elaeagnaceae)	leaves and twigs	acetone	HaCaT	N-[2-(5-hydroxyl-1H- indol-3-yl)ethyl]-butanamide, kaempferol-3-O-β-d-xylopyranosyl(1→2)-β-d-galactopyranoside-7-O-α-l-rhamnopyranoside, kaempferol-3-O-β-d-galactopyranoside-7-O-α-Lrhamnopyranoside, kaempferol-3-O-α-l-rhamnopyranosyl(1→6)-β-d-galactopyranoside-7-O-α-l-rhamnopyranoside, kaempferol-3-O-β-d-xylopyranosyl(1→2)-β-d-galactopyranoside	-	stimulate proliferation	[247]
*Annona crassiflora* Mart. (Annonaceae)	seeds	aqueous	HaCaT	catechin, epicatechin, rutin, quercetin, naringenin, protocatechuic acid, 4-hydroxybenzoic acid, vanillic acid, chlorogenic acid, caffeic acid, p-coumaric acid, ferulic acid	-	stimulate migration	[248]
*Combretum mucronatum* Schumach. & Thonn. (Combretaceae)	leaf	aqueous	NHEKs	epicatechin, procyanidinB2, vitexin and isovitexin	-	stimulate migration and differentiation	[249]
*Achillea asiatica* Serg. (Asteraceae)	aerial part	ethanolic	HaCaT	chlorogenic acid, schaftoside, quercetin-3-O arabinosyl(1→6)glucoside, apigenin-7-O-glucoside, luteolin, and apigenin	Induction: β-catenin, Akt	stimulate migration	[250]
*Moringa oleifera* Lam. (Moringaceae)	leaves	aqueous	NHEKs	vicenin-2, chlorogenic acid, gallic acid, quercetin, kaempferol, rosmarinic acid and rutin	-	stimulate migration and proliferation	[251]
*Plantago australis* Lam. (Plantaginaceae)	leaves	ethanolic	HaCaT	verbascoside	-	stimulate migration	[252]
*Aegle marmelos* (L.) Corrêa (Rutaceae)	flower	ethanolic	HaCaT	cineol, aegelin, cuminaldehyde, luvangetin, 1-hydroxy-5,7-dimethoxy-2-naphthalene-carboxaldehyde, eugenol	-	stimulate migration	[253]
*Boerhavia diffusa* L. (Nyctaginaceae)	leaves	methanolic	HaCaT	caffeic acid, ferulic acid and D-pinitol	-	stimulate migration	[254]
*Annona reticulata* L. (Annonaceae)	leaves	ethanolic	HaCaT	quercetin and β-sitosterol	Increased: VEGF and Akt	stimulate migration and proliferation	[255]
*Centella asiatica* (L.) Urb. (Apiaceae)	whole plant	methanolic	HaCaT	asiaticoside	-	stimulate migration	[256]
*Afgekia mahidoliae* B. L. Burtt & Chermsir. (Fabaceae)	leaves	chloroform/methanol	HaCaT	kaempferol-3-O-arabinoside, kaempferol-3-O-glucoside, and kaempferol-3-O-rutinoside,	Induction: filopodia and lamellipodia formation, Akt	stimulate migration	[257]
*Aloe vera* (L.) Burm.f. (Asphodelaceae)	leaves	aqueous	HPEKs	anthraquinones	Induction: β1-, α6-, β4-integrin, and E-cadherin expression	stimulate migration	[258]
*Aristolochia bracteolata* Lam. (Aristolochiaceae)	leaves	methanolic	HaCaT	quercetin	-	stimulate migration	[259]
*Stellera chamaejasme* L. (Thymelaeaceae)	aerial parts	ethanolic	HaCaT	daphnin, daphnetin-8-O-glucoside, daphnetin, rutarensin, isoquercitrin, chamechromone and daphnoretin	Induction: β-catenin, ERK and Akt	stimulate migration	[260]
*Polygonum aviculare* L (Polygonaceae)	whole plant	ethanolic	HaCaT	quercitrin hydrate, caffeic acid, and rutin	Induction: Wnt/β-catenin signaling	stimulate migration	[261]
*Hypericum carinatum* Griseb. (Hypericaceae)	aerial parts	n-hexane	HaCat cells	uliginosin A, japonicin A, uliginosin B, hyperbrasilol B, and the three benzopyrans, that is, 6-isobutyryl-5,7-dimethoxy-2,2-dimethyl-benzopyran, 7-hydroxy-6-isobutyryl-5-methoxy-2,2-dimethyl-benzopyran, and 5-hydroxy-6-isobutyryl-7-methoxy-2,2-dimethyl-benzopyran	-	stimulate migration	[262]

## Data Availability

Not applicable.
